# Preparation and Efficacy of Microemulsion Carvacrol-Based Fruit and Vegetable Cleaner and Its Application on Cherry Tomatoes

**DOI:** 10.3390/foods14020152

**Published:** 2025-01-07

**Authors:** Yanshuo Wang, Dianjun Sun, Yinghan Zhang, Yichong Zhou, Ruyi Jin, Xiaoli Peng, Jian Li

**Affiliations:** 1 China Food Flavor and Nutrition Health Innovation Center, Beijing Technology and Business University, Beijing 100048, China; 2 College of Food Science and Engineering, Northwest A&F University, Yangling 712100, China; 3 Key Laboratory of Green and Low-Carbon Processing Technology for Plant-Based Food of China National Light Industry Council, Beijing Technology & Business University (BTBU), Beijing 100048, China

**Keywords:** carvacrol, fruit and vegetable cleaner, antimicrobial, pesticide, cherry tomatoes

## Abstract

Carvacrol, a natural plant compound with antibacterial, antioxidant, and various biological activities, serves as the basis for developing a micro-emulsion fruit and vegetable cleaner. The study found that carvacrol demonstrated a minimum inhibitory concentration (MIC) ranging between 0.25 and 0.5 mg/mL against four foodborne pathogenic bacteria and three spoilage fungi. The formulated cleaner, containing 67 mg/mL of carvacrol, demonstrated superior characteristics (a particle size of 228 nm, an absolute zeta potential of 21.4 mv, and a stability coefficient of 91.2%). Remarkably, the cleaner remained stable when stored at room temperature for at least 3 months. Its efficacy against pesticides ranged from 76% to 91%. The cleaning effectively inhibited microbial colonies and the decay rate of cherry tomatoes during storage at 4 °C. Furthermore, the cleaning treatment was found to minimize changes in color and hardness. Overall, this study provides evidence that a fruit and vegetable cleaner based on carvacrol enhances the safety of the food industry effectively.

## 1. Introduction

Fruits and vegetables are vital sources of nutrition for human health. Many countries have established dietary guidelines to promote the consumption of fruits and vegetables [1]. The Food and Agriculture Organization of the United Nations further recommends a daily intake of at least 400 g of fruits and vegetables [2]. However, fresh fruits and vegetables carry the risk of containing foodborne pathogenic microorganisms and pesticide residues, thereby posing a potential threat to the food safety [3].

Harmful microorganisms found in fruits and vegetables, such as pathogenic bacteria and spoilage microorganisms, can lead to both food spoilage and foodborne diseases. The main pathogens causing foodborne diseases include *Listeria monocytogenes*, *Salmonella enterica* subsp. *enterica serovar* Typhimurium, *Staphylococcus aureus*, *Escherichia coli O157: H7*, etc. [4]. Fruit and vegetable spoilage is commonly caused by certain spoilage microorganisms, such as bacteria, yeasts, and molds [5]. The direct consequences of fruit and vegetable spoilage include causing social and economic losses. Additionally, it poses a significant threat to human health, leading to public health hazards [6].

Pesticide residues in fruits and vegetables constitute a comprehensive term referring to pesticides, their toxic metabolites, degradation products, and reaction impurities that persist on the surfaces of these foods following pesticide application [7]. The escalating global utilization of pesticides is paralleled by an increase in pesticide residues detected in fruits and vegetables [8]. According to the Ministry of Agriculture and Rural Affairs of China, the percentage of vegetables with pesticide residues surpassing legal limits was 3% in 2017 [9]. Despite the efficacy, long-lasting action, low toxicity, and significant potential for improving crop yield, the residues of broad-spectrum fungicides in agricultural production can pose health risks to humans [10].

Fruit and vegetable cleaner, valued for its antibacterial and pesticide residue removal properties, has become essential in people’s daily lives [11]. Microemulsions, characterized by their simple preparation, extremely low interfacial tension, high solubility, and outstanding physical stability, have seen extensive use in various fields in recent years [12]. Certain components found in essential oils from plants exhibit remarkable antibacterial and pesticide removal properties. However, their utility is limited by their insolubility in water and volatility [13,14]. Hence, creating a stable microemulsion of such components has the potential to significantly improve their utilization rate.

Carvacrol (C_10_H_14_O, 5-isopropyl-2-methylphenol) is an important phenolic metabolite extracted from oregano and thyme oils [15]. This substance can disrupt the cell membranes of bacteria, prevent biofilm formation, and demonstrate powerful antibacterial activity against a wide range of microorganisms [16,17]. The consumption of this monoterpenoid substance has been related to its antimicrobial, antioxidant, and antiseptic properties [18,19].

The non-toxic, environmentally friendly, and highly safe nature of carvacrol, coupled with its excellent antibacterial and freshness-preserving properties, position it as a promising alternative to chemical fungicides for controlling diseases affecting fruits and vegetables [20]. Carvacrol has been approved as a food additive in both the United States and Europe for several years. As a food additive, carvacrol may be used to impart flavor or provide antimicrobial properties to certain food products [21]. The approval of carvacrol as a food additive not only highlights its safety for consumption but also recognizes its potential benefits in preserving and enhancing the safety of daily chemical and food products.

The specific objectives of the study were to develop an environmentally friendly micro-emulsion fruit and vegetable cleaner using carvacrol. The newly developed cleaner should demonstrate effective antibacterial properties, a prolonged storage period, and the efficient removal of common pesticide residues. Furthermore, the storage quality of cherry tomatoes cleaned with this particular cleaner was evaluated.

## 2. Materials and Methods

### 2.1. Materials

Carvacrol was purchased from Shanghai Maclin Biochemical Technology Co., Ltd.(Shanghai, China) *E. coli O157:H7* (700728), *Staphylococcus aureus* (6538P), *Salmonella enterica* subsp. *enterica serovar* Typhimurium (13311), *Listeria monocytogenes* (19111), *Penicillium funiculosum* (36839), *Aspergillus Niger* (6275), and *Saccharomyces cerevisiae* (9763) were bought from the American Type Culture Collection (ATCC). Tryptone soya agar (TSA), trypticase soy broth (TSB), and potato dextrose agar (PDA) were purchased from Beijing Land Bridge Technology Co., Ltd. (Beijing, China) The alkaline phosphatase (AKP enzyme) test kit was purchased from Beyotime Biotechnology (Beijing, China). Tween 80, pan 20, polyglycerol fatty acid ester, alkyl polyglycoside C10-C16 (APG), sodium citrate, and xanthan gum were purchased from Aladdin. Imidacloprid, pyrimethil, pyrazolium, amamectin benzoate, and carbendazim were all purchased in the National Standard Material Center. All other chemicals were of analytical reagent grade and were obtained from Beijing Chemical Works (Beijing, China).

### 2.2. Antimicrobial Experiment

#### 2.2.1. Minimum Inhibitory Concentration (MIC) Determination

The frozen bacteria strains stored in −80 °C were inoculated in TSA medium and cultured at 37 °C for 12 h. A single colony was selected and inoculated into a 50 mL conical tube containing TBS medium, before being incubated at 37 °C for 12 h. After incubation, the bacterial suspension was centrifuged at 5000× *g* and 4 °C. The bacterial cells were washed with three PBS solution (pH = 7.2) cycles, and then the bacteria were centrifuged and suspended with the TSB to a concentration of 5 × 10^4^ CFU/mL. A volume of 8 mL bacterial suspension was added into the tubes, and a series of carvacrol concentrations (0, 0.0.1, 0.05, 0.15, 0.5, 0.75, 1.0, 2.5, and 5 mg/mL) were added to each tube. All test tubes were cultured in a 37 °C incubator for 24 h, and the optical density OD_600 nm_ values were detected with a spectrophotometer. When the bacterial inhibition rate exceeded 90%, the concentration of carvacrol was determined as an MIC value.

The MIC values of *Penicillium funiculosum*, *Aspergillus Niger*, and *Saccharomyces cerevisiae* were determined using the Oxford cup method. Approximately 20 mL of PDA medium was poured into a sterile Petri dish, after cooling and solidifying, and the spore suspension was evenly spread on the PDA medium. Three Oxford cups were evenly placed in each Petri dish, and 200 μL of carvacrol solution with a series of concentration gradients was added into the Oxford cup. After 48 h of culture at 26 °C, the diameter of the inhibition zone was measured. The MIC value was the concentration of carvacrol when the inhibition rate reached 90%.

#### 2.2.2. Cell Wall Permeability and Intracellular ROS Detection

Compared to eukaryotes, prokaryotes such as bacteria have stronger cell permeability. The cell wall permeability of the bacteria was assessed using the method of [22] with minor revisions. In brief, bacterial cells diluted with PBS (1 × 10^8^ CFU/mL) were treated with carvacrol at a concentration equivalent to 0 (control), 1/2 MIC, MIC, and 2 MIC and then incubated at 37 °C. Then, 1 mL of the bacteria suspension was taken to detect the cell wall permeability at 30, 60, 90, 120, and 150 min using the AKP enzyme activity kit.

The intracellular ROS levels were detected using DCFH-DA fluorescent probe according to the method of [23]. The bacterial suspensions (1 × 10^8^ CFU/mL, in PBS) from each treatment (0, 1/4, 1/2 MIC, and MIC) were incubated at 37 °C for 2 h. Samples were incubated with 5 μmol/L DCFH-DA at 37 °C for 10 min, and the fluorescence intensities were measured using a multimode microplate reader platform (InfiniteTM M200 PRO Spark, Tecan company, Männedorf, Switzerland) with excitation and emission wavelengths of 488 nm and 525 nm, respectively.

### 2.3. Cleaner Preparation

#### 2.3.1. The Determination of the Proportion of Surfactant

The response surface method was employed to optimize the quantity of mixed surfactant for a 6.7% carvacrol aqueous solution. Following a pre-experiment single-factor test, three levels of Tween 80, Span 20, and polyglycerol fatty acid ester were selected as low, medium, and high (Supplementary Table S1).

#### 2.3.2. The Determination of the Optimal Formula

The optimal formula was determined using an orthogonal experimental design, focusing on the influence of three specific factors on the stability of the cleaning agent. In this investigation, the L9 (34) orthogonal table was deemed suitable. The evaluation of the stability was based on key parameters, including pH, viscosity, and foaming. To evaluate stability, scores ranging from 1 to 10 points were assigned on a scale. Ultimately, the optimal formula was identified through thorough analysis, and the details of the orthogonal design for the formula are presented in Table 1.

#### 2.3.3. Preparation of the Cleaner

Xanthan gum (0.1 mg/mL) was first dissolved in ultrapure water and stirred (10 r/s, 50 °C) until complete dissolution was achieved. Subsequently, sodium citrate (10 mg/mL) and APG (30 mg/mL) were added, and the surfactants, namely Tween 80 (59 mg/mL), Span 20 (37 mg/mL), and polyglycerol fatty acid ester (25 mg/mL), were introduced in succession after the complete dissolution of each component, respectively. After setting the temperature to 25 °C, carvacrol was introduced, and the mixture was stirred for a duration of 20 min and then the mixture underwent ultrasonication (200 W) for 2 h. It is important to note that the container remained sealed throughout the entire preparation process.

### 2.4. Characterization Analysis

#### 2.4.1. Thermal, Freeze–Thaw, and Storage Stability

For thermal stability measurements, the samples underwent heating in a water bath at 25, 50, 75, and 100 °C for 30 min, respectively. Then, the samples were cooled down for 1 h to room temperature before further analysis. For freeze–thaw stability analysis, the cleaner was poured into a glass container and stored at −20 °C for 24 h. Subsequently, the samples were subjected to 3 cycles of thawing at room temperature. Properties were then assessed after each thawing cycle [24]. For storage stability measurement, the cleaner was stored at two different temperatures, 4 °C and 25 °C, for a duration of 1 year. The cleaner was sampled every 15 days to detect the properties.

#### 2.4.2. Z-Average Diameters, Zeta-Potential, and PDI (Polydispersity)

The Z-average diameters, zeta-potential, and PDI of the cleaner were determined using a Zetasizer Nano-ZS (ZEN 3600, Malvern, UK). The samples were diluted 300-fold with ultrapure water to eliminate multiple scattering effects. The He-He was used as a laser and the ultrapure water was used as the dispersant. The instrument parameters were set as follows: a wavelength of 633 nm, a scattering angle of 173°, and a refraction index of 1.40 [25].

#### 2.4.3. Stability Coefficient (R)

The R of the cleaner was measured, as reported by [25], with minor revisions. In brief, the cleaner was diluted 100-fold with ultrapure water and then centrifuged for 15 min at 3000× *g*. The subnatant was collected and diluted 100-fold using ultrapure water. The absorbance was determined at 410 nm. The R was expressed as follows:

$$R\left(\%\right)=\frac{A_{1}}{A_{0}}\times 100\%$$

where A_0_ was the absorbance before centrifugation and A_1_ was the subnatant absorbance after centrifugation.

### 2.5. Antimicrobial Effect and Pesticide Residue Removal Ability of the Cleaner

#### 2.5.1. Antimicrobial Testing

The antimicrobial effect was measured using the Oxford cup method. A total of 4 groups were set up: control (diluted water), carvacrol, commercial fruit and vegetable cleaner, and carvacrol cleaner. Both cleaners were diluted 500-fold. The bacterial plates were settled at 37 °C for 24 h, while the fungi plates were placed at 26 °C for 72 h. The size of the inhibition zones was detected after culture.

#### 2.5.2. Pesticide Residue Removal Ability

According to the GB/T24691-2009 standard, 5 sorts of pesticide emulsions were prepared, and celery, beans, leeks, and cherry tomatoes were selected as test samples [26]. The fruit and vegetable samples were immersed in the pesticide solution for 30 min, and then ventilating-dried for 24 h. The samples were then divided into 4 groups. Subsequently, samples were soaked in the solutions (distilled water as the control, carvacrol, 1:500 commercial cleaner, and 1:500 carvacrol cleaner) for 30 min (a sample-to-solution ratio of 1:2), stirred three cycles for 3 min each cycle during the period. After that, the samples were immersed in sterile water (a sample-to-solution ratio of 1:2) 3 cycles, with 5 min each cycle, and then dried for 2 h and sampled.

The pesticide residual was analyzed using the LC-MS method. Briefly, 25 g samples were mixed with 50 mL of acetonitrile and then homogenized for 2 min. A volume of 40 mL filtrate was collected and added into 100 mL 5% NaCl solution and kept for 30 min after violent oscillation. Then, 1 mL of supernatant was mixed with 1 mL of methanol aqueous solution (1:1), and then filtered using the organic filter membrane for LC-MS (LCMS-2050, Shimadzu, Japan) analysis.

#### 2.5.3. Residual Amount After Washing

The APG residual was detected, according to the National Standard of China (GB/T 24691-2022) [27]. Briefly, the absorbance of the solution after cleaning beans with the carvacrol cleaner and the commercial cleaner was measured at 625 nm with a spectrophotometer.

### 2.6. The Storage Effect of Cherry Tomatoes After Cleaning

#### 2.6.1. Sample Treatment

Freshly harvested ‘Bijiao’ cherry tomatoes were purchased at Yangling and then immersed in different solutions (distilled water as control, carvacrol, 1:500 commercial cleaner, and 1:500 carvacrol cleaner) for 30 min (a sample-to-solution ratio of 1:2), and stirred three times for 3 min each time during the period. After that, the samples were immersed in sterile water (a sample-to-solution ratio of 1:2) 3 times, with 5 min each time. After being air-dried, the cherry tomatoes were packaged and storage at 4 °C for later measurement.

#### 2.6.2. Weight Loss, Rot Rate, Sensory Score, and Microbial Evaluation

Weight loss was expressed as the percentage decrease in the final weight of the cherry tomatoes to the initial weight, while the rot rat was expressed as the percentage of the number of rotten fruit to the total amount of the samples treated [28].

Five graduate students from the School of Food Science and Engineering were selected as members of the sensory evaluation panel. Before the sensory evaluation began, the team members were trained to ensure that the evaluation team members were clear about the scoring rules. Sensory attributes, including color, taste, odor, texture, and overall acceptance, were evaluated using the 10–0-point category test, where 8–10 is excellent, i.e., extremely fresh; 6–8 is very good, i.e., slightly dark and slightly wrinkly; 4–6 is good, i.e., dark color, soft, and serious wrinkles; 2–4 indicates limited usability, i.e., dark color with yellow spots and severe wrinkles; and 0–2 is extremely poor, i.e., mold, peel collapse, sour smell, and spoiled.

The microbiological analysis of the cherry tomatoes was performed with reference to GB4789.2-2016 [29]. Samples (25 g) were placed into a sterile homogenized bag and 225 mL of normal saline was added. The homogenized solution was diluted in 10-fold increments, and 1 mL of the diluent was absorbed and added into the plate counting agar medium. After 48 ± 2 h at 36 °C, the colonies on the plate were counted, and the results were expressed as log CFU/g.

#### 2.6.3. Color

The color value of the samples was assessed with a Ci7600 colorimeter (Ailise, Shanghai, China). The values of L* (lightness), a* (redness-greenness), and b* (blueness–yellowness) of 10 fruits were measured each time. The Hue angle (H*) (sexagesimal degrees) was calculated with the following equation:

$$H*=\arctan(\frac{b*}{a*})$$

#### 2.6.4. Hardness, Total Soluble Solids (TSSs), Titratable Acidity (TA), and Vitamin C (Vc) Analysis

The hardness was determined using TA. An XT PLUS texture analyzer (Stable Micro System, Godalming, UK) was used, as described by [30]. A probe with a 8 mm diameter was used to penetrate the fruit to a depth of 10 mm at a speed of 10 mm/s. The results are presented as N/cm^2^.

A hand-held digital refractometer (SW32A, Suwei, Guangdong, China) was used to determine the content of TSSs. Next, 30 g cherry tomatoes were crushed to extract juice and the results were expressed as percentages [28].

The TA was determined via acid-base titration, as described [31], and the results were expressed according to the volume of NaOH used.

The content of Vc was determined via 2,6-dichlorophenol titration, according to the National Standard of China (GB 5009.86-2016) [32]. The Vc concentration was calculated according to the titration volume of 2,6-dichlorophenol and expressed as mg/100 g of fresh weight.

### 2.7. Statistical Analysis

Experiments were carried out in triplicate unless otherwise specified, and the data were described as the mean ± standard deviation. One-way analysis of variance (ANOVA) and Duncan’s multiple test were used to test for statistical significance. Differences of *p* < 0.05 were considered statistically significant.

## 3. Results and Discussion

### 3.1. Antimicrobial Effects of Carvacrol

Foodborne pathogenic bacteria and spoilage fungi are the primary contributors to the loss of the nutritional and economic value of fresh fruit and vegetable products [33,34]. As indicated in Table 2, the MIC value of carvacrol against *Staphylococcus aureus* and *Listeria monocytogenes* was 0.5 mg/mL, while the MIC against *E. coli O157:H7* and *Salmonella enterica subsp. enterica serovar* Typhimurium was 0.25 mg/mL. Results from the Oxford Cup experiment confirm MIC values of carvacrol against *Penicillium funiculosum*, *Saccharomyces cerevisiae*, and *Aspergillus niger* as 0.25 mg/mL, 0.25 mg/mL, and 0.50 mg/mL, respectively. These findings highlight the significant antimicrobial effects of carvacrol against both common foodborne pathogenic microorganisms and spoilage microorganisms. The findings are generally consistent with the results on bacteria, mold, and yeast, even though some strains were inconsistent (in previously published literature studies, the MIC of carvacrol ranged from 0.015 to 0.03% *v*/*v*) [35,36]. Consequently, carvacrol showed promise for use in the development of fruit and vegetable cleaner products.

**Figure 1:**
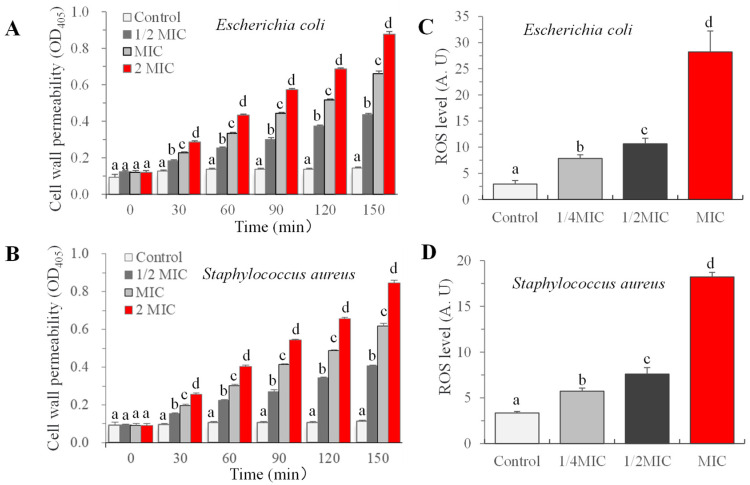
The effect of carvacrol on cell wall permeability (**A**,**B**) and intracellular ROS levels (**C**,**D**). Bars (mean ± std dev, *n* = 3) with different letters indicate mean values that are significantly different (*p* < 0.05).

Under typical conditions, AKP, an enzyme located between cell wall and cell membrane, is difficult to detect in the culture solution, but when the cell wall is destroyed, AKP enzyme leakage occurs [37]. The study reveals that upon treatment with carvacrol, the levels of AKP enzyme in bacterial solutions of *Staphylococcus aureus and E. coli* increased proportionally with both the treatment time and the concentration, as illustrated in Figure 1A,B. The intracellular ROS levels of microorganisms after 2 h of carvacrol incubation were further detected. The results showed that with an increasing in carvacrol concentration, the intracellular ROS levels of bacteria exhibited an upward trend. Specifically, at an MIC concentration, the ROS levels of *E. coli O157:H7* and *salmonella* were 9.57 times and 5.44 times higher than the control, respectively (Figure 1C,D). These results were consistent with the previous conclusion, suggesting that carvacrol damaged the structural integrity of bacteria and elevated ROS levels [19,38]. Indeed, carvacrol exhibited a potent destructive impact on both Gram-positive and Gram-negative bacteria, promoting intracellular ROS production and eventually leading to cell damage.

### 3.2. The Preparation of Carvacrol-Based Fruit and Vegetable Cleaner

#### 3.2.1. Response Surface Optimization Results and Analysis

Following the Box–Behnken central composite design principle, regression analysis was conducted using the solubility score of carvacrol as the dependent variable. Parameters such as the stratified volume after solution stabilization and the emulsified area within the terpolymer phase diagram were considered in the analysis. Supplementary Table S2 displays the design and results of Box–Behnken, and the outcomes from the 17 groups in Table 3 were further were fitted using Design-Expert 7.0. The quadric function expression detailing the influence of factors A, B, and C on the carvacrol solubility score (Y) was expressed as the following: Y = 86.32 + 4.42A + 3.85B + 3.12C − 2.94AB − 2.47AC − 1.44BC − 6.23A^2^ − 4.89B^2^ − 9.25C^2^. Both *R*^2^ (0.9011) and *R*^2^*_Adj_* (0.9494) were close to 1, indicating the model’s accuracy in explaining the variation in the solubility score of carvacrol. The equation fitted well, allowing for the prediction of the carvacrol solubility score. Further correlation analysis is shown in Supplementary Table S3, and the order of influence of three factors on the solubility score of carvacrol was determined to be A (Tween 80) > B (Span 20) > C (polyglycerol fatty acid ester).

**Figure 2:**
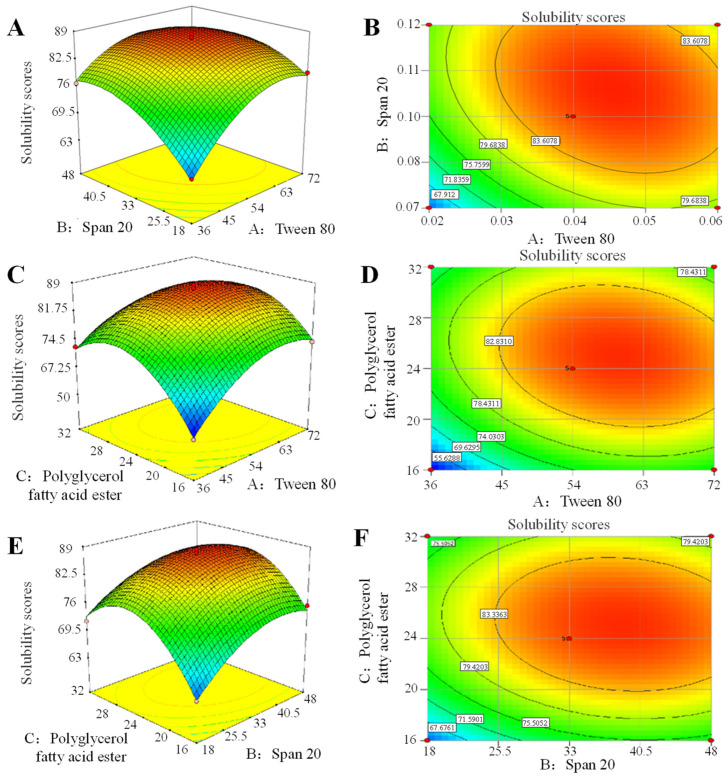
The response of the surface and contour of the three factors (Tween 80, Span 20, and polycerol fatty acid ester) to the carvacrol solubility score. (**A**,**B**) The response of the surface and contour of Tween 80 and Span 20 to the carvacrol solubility score. (**C**,**D**) The response of the surface and contour of Tween 80 and polyglycerol fatty acid ester to the carvacrol solubility score. (**E**,**F**) The response of the surface and contour of Span 20 and polyglycerol fatty acid ester to the carvacrol solubility score.

According to the response surface methodology (RSV), the influence of various factors on the solubility score of carvacrol and the interaction among these factors can be intuitively seen [39]. As evident in (Figure 2A,B), the interaction between the two factors, Tween 80 and Span 20, was notably significant. In the corresponding surface curve for the solubility of carvacrol, there was an initial rapid increase followed by a tendency to plateau. Additionally, the contours were found to exhibit an almost elliptical shape, emphasizing the substantial interplay between Tween 80 and Span 20 in influencing the solubility of carvacrol. Similar conclusions can be drawn from Figure 2C,D, as there were significant interactions between Tween 80 and the amount of polyglycerol fatty acid ester added, as well as between Span 20 and the amount of polyglycerol fatty acid ester added. These results supported the idea that increasing the amount of T80, S20, and polyglycerol fatty acid ester can improve the response value, but an excessively high addition amount is not conducive to improving the solubility score.

Design-Expert 7.0 analysis was further used to optimize the solubility of carvall (score 87.64), and the following optimal conditions were obtained: Tween 80—58.73 mg/mL, Span 20—37.47 mg/mL, and polyglycerol fatty acid ester—24.89 mg/mL.

#### 3.2.2. Optimal Formula Orthogonal Experiment

The orthogonal test was then employed to identify the optimal formula. As indicated in Supplementary Table S3, the impact of the three factors on the stability score followed the following order: A > B > C. The optimum ratio was determined to be A1B2C1 (stability score 9.5), signifying the xanthan gum at 0.1 mg/mL, the sodium citrate at 10 mg/mL, and the APG at 3%. Analysis of Supplementary Table S5 further revealed that xanthan gum and sodium citrate significantly influenced the stability of the cleaner *(p* < 0.05), while APG showed no statistically significant effect (*p* > 0.05).

#### 3.2.3. Quality Analysis of the Cleaner

**Figure 3:**
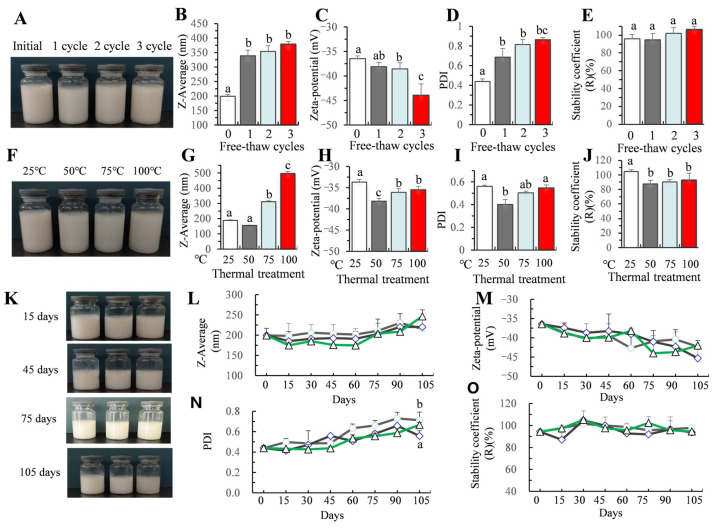
The effect of free–thaw (**A**–**E**) and thermal treatment on the stability (**F**–**J**) of the cleaner and the storage stability (**K**–**O**) of the cleaner. Bars (mean ± std dev, *n* = 3) with different letters indicate mean values that are significantly different (*p* < 0.05).

As shown in Figure 3A, after three freeze–thaw cycles, the appearance of the cleaning agent had no obvious change. The particle size increased from 199.4 nm to 339 nm after one freeze–thaw, and the more freeze–thaw cycles, the greater the increase in particle size, but the difference between freeze–thaw cycles was not significant (Figure 3B). The change in particle size during the first freeze–thaw period is the most severe, which may be due to the mechanical expansion force of ice crystal particles generated by freezing, resulting in the destruction of microemulsion structure. With the increase in the number of cycles, although the potential of the cleaning agent decreased significantly and the PDI value increased continuously, the stability did not change significantly within 3 cycles of freezing and thawing (Figure 3C,E), which was consistent with the result of no obvious aggregation in appearance.

The thermal stability experiment showed that with an increase in temperature, the particle size of the cleaning agent increased, and the potential, PDI, and stability decreased slightly (Figure 3F,G). Excessive temperature treatment may accelerate the hydrolysis of the emulsifier ester bond, and the emulsifying effect of T80 was slightly reduced. However, there was no caking and demulsification during the whole heating process, and the solution basically remained stable. In conclusion, carvacrol still has high stability in different heat treatments. Our results were consistent with the conclusions that the average particle size of the carvacrol nanoemulsion prepared with Tween 80 as the emulsifier and distilled water as the water phase was stable at 25 °C, 60 °C, and 100 °C, with an average size ranging between 180 nm and 200 nm [40].

The storage stability test can provide reference for the storage conditions and expiration dates of the fruit and vegetable cleaner. Within a selected temperature range between 4 °C and 37 °C, the cleaning agent formed a stable dispersion system, exhibiting no sign of precipitate, flocculate, agglomerate, phase separation, and other unstable phenomena during storage (Figure 3K). After storage at 4 °C, 25 °C, and 37 for 4 months, there was no significant difference observed in average particle size, zeta-potential, and PDI, and the stability coefficient of the cleaner showed no significant difference (Figure 3L,O). In conclusion, the carvacrol cleaning agent showed good storage stability, and it was recommended to be stored at room temperature for economic consideration.

### 3.3. Antimicrobial Effect and the Removal Efficacy of Pesticide Residue

#### 3.3.1. Antimicrobial Effect

The antibacterial efficacy of the cleaning agent was further assessed against seven types of microorganisms. Generally, the order of antibacterial zone diameters observed was as follows: carvacrol-based cleaner > carvacrol > commercial cleaner. Notably, carvacrol-based cleaner exhibited the most potent inhibitory effects on *Penicillium funiculosum* and *Aspergillus Niger*, with antibacterial zones reaching 42.3 and 39.3 cm, respectively. This suggested its effectiveness in preventing fruit and vegetable spoilage. Compared to carvacrol alone, the high antibacterial effect of the cleaner should be related to the large amount of hydrophilic groups contained in xanthan gum; therefore, carvacrol slowly volatilizes and exerts antibacterial effects more continuously and effectively [41,42].

#### 3.3.2. The Removal Efficacy of Pesticide Residue

**Figure 4:**
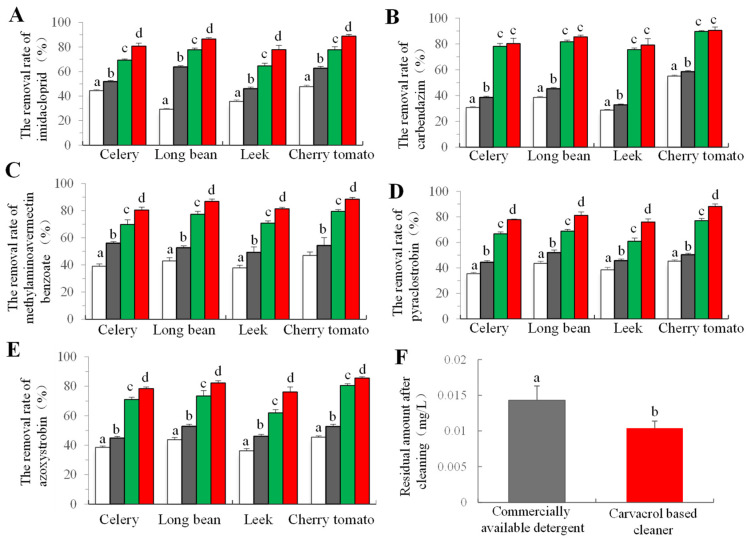
Residue of the pesticide and residue of the cleaning agent after washing. The removal of imidacloprid (**A**), carbendazim (**B**), methylaminoavermectin benzoate (**C**), pyraclostrobin (**D**), and azoxystrobin (**E**) from celery, bean, leek, and cherry tomatoes samples among different cleaning groups. The residual amount of APG residue after cleaning in beans (**F**). Bars (mean ± std dev, *n* = 3) with different letters have mean values that are significantly different (*p* < 0.05).

Pesticide residues employed in the cultivation of fruits and vegetables have the potential to infiltrate the human body through the food chain, thereby posing potential risks to human health [43]. Encouragingly, four washing methods, including distilled water, demonstrated a certain efficacy in removing residues of five distinct pesticides from fruits and vegetables (Figure 4A–E). Particularly noteworthy was the significantly superior pesticide removal performance of the carvacrol cleaner group compared to other treatment groups. Specifically, the clearance rate of the self-made cleaning agent for carbendazim in cherry fruit exceeded 90%, while the removal rate of other tested pesticides ranged between 75% and 90%. Although the cleaning effect of carvacrol alone surpassed that of the clean water group, the cleaning efficiency of the both cleaning agents was significantly higher than that of the other two groups, indicating that the surface expander and other components of the cleaning agent played a greater role in the removal of agricultural residues.

The residual amount of APG after washing soybean with fruit and vegetable detergent was investigated. Findings revealed that the residual amount of carvacrol fruit and vegetable detergent after washing was 10.3 μg/mL and that of the commercial fruit and vegetable detergent group was 14.2 μg/mL (Figure 4F). The residual amount of the two groups was significantly different (*p* < 0.05).

### 3.4. Storage Effect of Cherry Tomatoes After Cleaning

#### 3.4.1. Sensory Score, Weight Loss, Rotting Rate, and Microbial Evaluation

**Figure 5:**
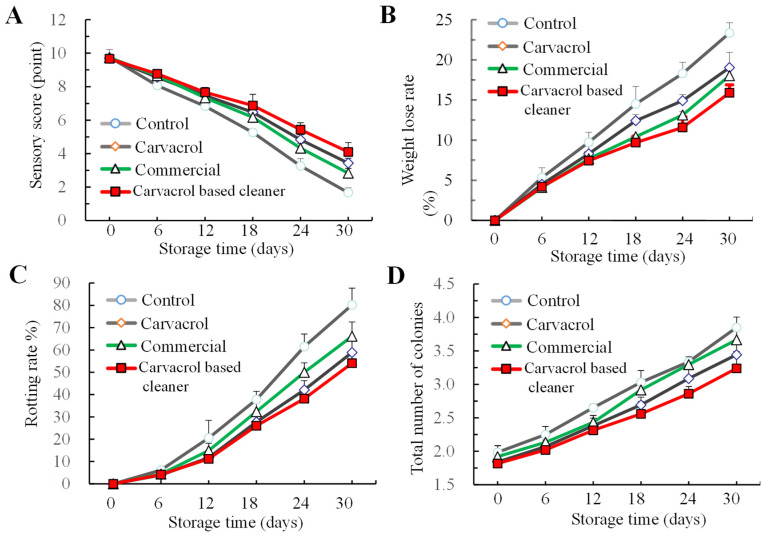
Changes in the sensory score (**A**), weight loss rate (**B**), rotting rat (**C**) and total number of colonies (**D**) in cherry tomato washed with the cleaners and storage for 30 days at 4 °C. Bars (mean ± std dev, *n* = 3) have mean values that are significantly different (*p* < 0.05).

To validate the efficacy of the cleaner, cherry tomatoes were chosen, washed, and subsequently stored at 4 °C. With the extension of storage time, the sensory scores of different treatment groups exhibited a downward trend, with the carvacrol group demonstrating the narrowest range of sensory scores (Figure 5A). After 12 days of storage, the weight loss rate of the control group was significantly different from that of the other groups. By the 30th day of storage, a weight loss of 23.4% in the control group and a weight loss of 15.9% in the self-made cleaning agent group were observed (Figure 5B).

The decay rate during storage serves as a direct indicator of storage efficacy. Throughout the storage duration, treatment with the carvacrol cleaning agent attenuated the decay rate of cherry tomatoes and the proliferation of total colony counts (Figure 5C,D). This indicated that the self-made cleaning agent can inhibit microbial growth during the storage process of cherry tomatoes post-cleaning, reduce decay, and extend the storage period, consequently improving edibility and safety. These observations align with previous findings on strawberries treated with carvacrol activity package, where carvacrol essential oil effectively controls the surface microorganisms and reduces the rot rate [44].

#### 3.4.2. Color

**Figure 6:**
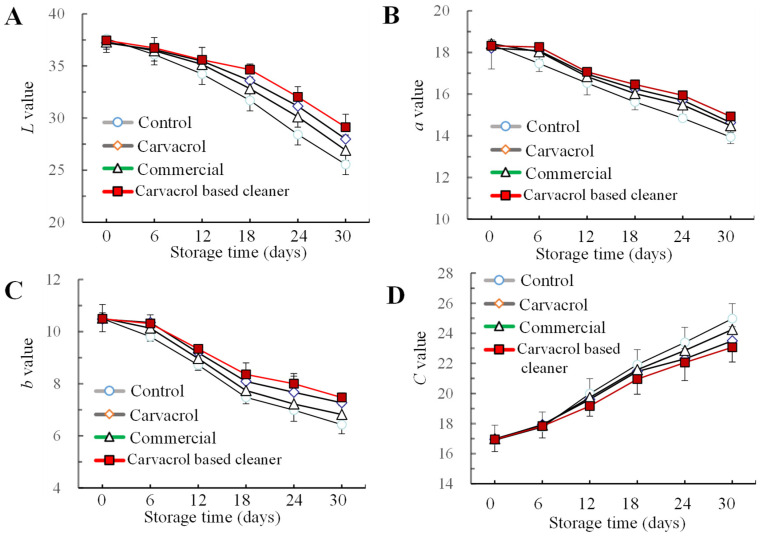
Changes in the L* (**A**), *a* (**B**), *b* (**C**), and *C* (**D**) values of cherry tomatoes washed with the cleaners and stored for 30 days at 4 °C. Bars (mean ± std dev, *n* = 3) indicating mean values that are significantly different (*p* < 0.05).

The color of fruits and vegetables serves as the most intuitive quality indicator for consumers [45]. During storage, the *L* value, *a* value, and *b* value of all groups of samples decreased, while the *C* value exhibited an increasing trend. By day 30, the *L* value (29.13), *a* value (14.93), and *b* value (8.62) in the self-made cleaning agent group were 14.0%, 7.1% and 16.2% higher than those in the control group, respectively (Figure 6A–C). The *C* value represented the combined effect of the *a* value and *b* value. In the initial 6 days of storage, the change in the *C* value among all groups showed no obvious difference and was about 17° (Figure 6D). Since the 12th day, the change in the *C* value of the self-made cleaning agent treatment group was significantly smaller than that of the other three groups (*p* < 0.05). The color attributes of cherry tomatoes were influenced by pigment content, particularly lycopene concentration [46]. The self-made cleaning agent treatment group had the most obvious effect on the fruit brightness decline of cherry tomatoes. These findings indicated that the carvacrol cleaning agent, especially carvacrol, may have delayed the change in the color of cherry tomatoes due to the degradation of pigment.

#### 3.4.3. Hardness, Total Soluble Solids (TSSs), Titratable Acidity (TA), and Vitamin C (Vc)

**Figure 7:**
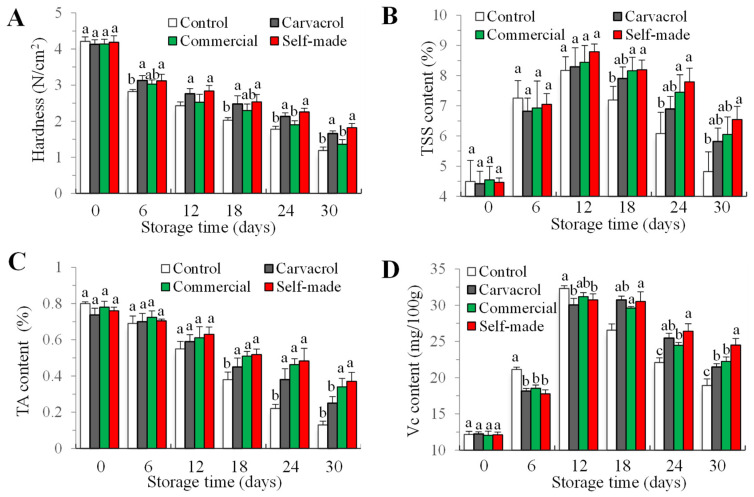
Changes in the hardness (**A**), total soluble solids (TSSs) (**B**), titratable acidity (TA) (**C**), and vitamin C (Vc) (**D**) content in cherry tomato washed with the cleaners and storage for 30 days at 4 °C. Bars (mean ± std dev, n = 3) with different letters indicate mean values that are significantly different (*p* < 0.05).

From the sixth day, the hardness of cherry tomatoes in control group significantly differed from that in the essential oil treatment group and carvacrol cleaner treatment group (*p* < 0.05). By day 30, the hardness of cherry tomatoes in the carvacrol cleaner group was 52.1% higher than that in the control group (Figure 7A). In addition, there was no significant difference between the self-made cleaning agent group and the oil treatment group, indicating that carvacrol played a pivotal role in slowing the softening of cherry tomatoes during storage, which may be attributed to the inhibition of microbial growth and the inhibition of enzyme activity related to the softening of fruits and vegetables.

TSS levels and TA were found to have a significant impact on the flavor and taste of cherry tomatoes. During storage, cherry tomatoes’ TSS levels grew gradually after storage until peaking on day 12. Following day 18, TSS levels in the control group were significantly lower than those in the other groups, while no significant difference was found between the other three groups (*p* < 0.05). The TSS content of the carvacrol cleaner group was maintained at the highest level throughout the storage period (Figure 7B). The control group’s TA content had significantly dropped, in contrast to the other groups, with a downward trend overall (Figure 7C). The inhibition effect of carvacrol cleaner on the decrease in the TSS content of cherry tomatoes was obviously better than that of the other two groups, and it was more beneficial to improve the quality of cherry tomatoes during storage. The change trend of TSS content and TA in this study matched [47]’s findings. In the middle and later stages of storage, cherry tomatoes consume a lot of nutrients in respiration and TSS content gradually decrease [48].

During the storage period of cherry tomatoes, the content of Vc in each group showed a trend of first increasing and then decreasing (Figure 7D). By day 6, there was a significant difference in the content of Vc in the control group (*p* < 0.05). Following a 24-day period of storage, a noteworthy distinction was observed in the Vc content of cherry tomatoes between the groups treated with commercial cleaner and those treated with carvacrol cleaner (*p* < 0.05). In the whole process of the decrease in the Vc content in cherry tomatoes, the carvacrol cleaner group demonstrated the most obvious inhibition effect on the decrease rate of Vc content. The level of Vc content in cherry tomatoes initially increased during storage and then continuously decreased, which may be caused by the respiration action, oxidative deterioration, and the rapid accumulation of carbon dioxide [30,49].

## 4. Conclusions

Fruit and vegetables that rot fungi and common foodborne pathogenic bacteria are both effectively inhibited by carvacrol. A decreased average diameter (228 nm) and greater stability at temperature treatment (≤75 °C), as well as storage over a wide temperature range (4–37 °C), for at least 105 d were demonstrated by the fruit and vegetable cleanser based on carvacrol (67 mg/mL). The self-made cleaning agent showed an obvious bacteriostatic effect, and the removal rate of pesticide in fruits and vegetables ranged between 76% and 91%, with a lower level of residual amount of APG (10 μg/L) after cleaning, making it a safer alternative. Cherry tomatoes, when cleaned with this specific cleaner and subsequently stored at 4 °C, demonstrated enhanced efficacy in reducing the total number of bacterial colonies on the fruit surface. Additionally, the treatment led to a decreased decay rate, minimized changes in color and hardness, and a slower decline in nutritional qualities. This study demonstrated that carvacrol-based fruit and vegetable cleaner improves the safety of the food industry effectively.

This experiment explored the application of carvacrol and developed a carvacrol fruit and vegetable cleaner, which contributed to the economic benefits of cherry tomatoes. Based on the research conclusions, future studies can use the excellent antibacterial activity of carvacrol to develop other products for the cleaning of agricultural products. It can also comprehensively explore the physiological activity of carvacrol, so that it can be applied in more fields.

## Figures and Tables

**Table 1:** Levels of liquefaction orthogonal.

Level	A	B	C
	Xanthan Gum (mg/mL)	Sodium Citrate (mg/mL)	APG (%)
1	0.1	0.05	3
2	0.3	0.1	5
3	0.5	0.2	7

**Table 2:** The minimum inhibitory concentration of carvacrol.

Microbial Species	Strain	MIC (mg/mL)
*E. coli* *O157:H7*	ATCC 700728	0.25
*Staphylococcus aureus*	ATCC 6538P	0.50
*Listeria monocytogenes*	ATCC 19111	0.50
*Salmonella enterica subsp. enterica serovar* Typhimurium	ATCC 13311	0.25
*Penicillium funiculosum*	ATCC 36839	0.25
*Aspergillus niger*	ATCC 6275	0.25
*Saccharomyces cerevisiae*	ATCC 9763	0.50

**Table 3:** The size of the inhibition zone of different fruit and vegetable cleaners on test bacteria and fungi (unit: mm).

	Control	Carvacrol Group	Commercial Group	Carvacrol-Based Cleaner
*E. coli O157:H7*	—	33.3 ± 0.34 ^b^	30.6 ± 0.21 ^a^	34.3 ± 0.22 ^c^
*Staphylococcus aureus*	—	24.7 ± 0.14 ^b^	21.7 ± 0.33 ^a^	25.4 ± 0.32 ^c^
*Salmonella* *enterica subsp. enterica serovar* Typhimurium	—	22.8 ± 0.37 ^b^	16.2 ± 0.62 ^a^	23.4 ± 0.28 ^b^
*Listeria monocytogenes*	—	27.3 ± 0.31 ^b^	18.9 ± 0.32 ^a^	28.3 ± 0.25 ^c^
*Penicillium funiculosum*	—	41.9 ± 0.46 ^b^	35.3 ± 0.47 ^a^	42.3 ± 0.38 ^b^
*Aspergillus niger*	—	38.3 ± 0.33 ^b^	34.7 ± 0.26 ^a^	39.3 ± 0.21 ^b^
*Saccharomyces cerevisiae*	—	26.9 ± 0.24 ^b^	24.3 ± 0.30 ^a^	27.2 ± 0.23 ^b^

Different letters indicate mean values that are significantly different (*p* < 0.05).

## Data Availability

The original contributions presented in this study are included in the article/supplementary material. Further inquiries can be directed to the corresponding authors.

## Supplementary Materials

The following supporting information can be downloaded at: https://www.mdpi.com/article/10.3390/foods14020152/s1, Table S1. Factors and levels of Box-Behnken design; Table S2. Results of response surface analysis; Table S3. The analysis results of regression and variance; Table S4. Orthogonal test results; Table S5. The variance analysis.
