# Supplementary material for: Preparation and Efficacy of Microemulsion Carvacrol-Based Fruit and Vegetable Cleaner and Its Application on Cherry Tomatoes
Source: Foods. 2025 Jan 7;14(2):152. doi: 10.3390/foods14020152 (PMC11764712; doi:10.3390/foods14020152)
Supplement: Supplementary file 1 [file foods-14-00152-s001.zip › foods-3324794-supplementary.pdf]

Supplementary Materials

Supplementary table S1. Factors and levels of Box-Behnken design

Factor	Level		
	-1	0	1
A: Tween 80（mg/ml）	36	54	72
B: Span 20（mg/ml）	18	36	48
C: polyglycerol fatty acid ester（mg/ml）	16	24	32

Supplementary table S2. Results of response surface analysis

Number	A（Tween 80）	B（Span 20）	C（Polyglycerol fatty acid ester）	Solubility score
1	-1	-1	0	64.48
2	1	-1	0	79.39
3	-1	1	0	76.86
4	1	1	0	80.03
5	-1	0	-1	60.42
6	1	0	-1	73.98
7	-1	0	1	72.64
8	1	0	1	76.32
9	0	-1	-1	63.68
10	0	1	-1	75.46
11	0	-1	1	71.78
12	0	1	1	77.79
13	0	0	0	84.58
14	0	0	0	83.30
15	0	0	0	87.71
16	0	0	0	88.38
17	0	0	0	87.62

Supplementary table S3. The analysis results of regression and variance

* indicates a significant difference at P<0.05); ** indicates significant difference at P<0.01.

Items	Square sum of	Degrees of freedom	Mean square	F-value	P-value	Significance
Model	1110.72	9	123.41	34.39	< 0.0001	**
A-Tween 80	156.01	1	156.01	43.47	0.0003	**
B-Span 20	118.70	1	118.70	33.08	0.0007	**
C-polyglycerol fatty acid ester	78.08	1	78.08	21.76	0.0023	**
AB	34.48	1	34.48	9.61	0.0173	*
AC	24.44	1	24.44	6.81	0.0349	*
BC	8.29	1	8.29	2.31	0.1723	
A^2	163.44	1	163.44	45.54	0.0003	**
B^2	100.86	1	100.86	28.11	0.0011	**
C^2	359.98	1	359.98	100.31	< 0.0001	**
Residual error	25.12	7	3.59			
Missing fit	5.06	3	1.69	0.34	0.8014	
Pure error	20.06	4	5.02			
Sum	1135.85	16				

The data in the supplementary table S3 showed that interaction terms AB and AC had significant effects on the solubility score of carvacrol (P< 0.05), while interaction terms BC had no significant effects on the solubility score of carvacrol (P> 0.05). Primary items A, B, C and secondary items A2, B2, C2 had significant effects on the solubility score of carvacrol (P < 0.001).

Supplementary table S4. Orthogonal test results

A: 1=1mg, 2=3 mg, C=5mg; B: 1=5 mg, 2=10 mg, 3=20 mg; C:1=3%, 2=5%, 3=7%

Test Number	A（Xanthan gum, mg）	B（Sodium citrate）	Blank column	C（APG）	Stability score（1~10）
1	1	1	1	1	6.5
2	1	2	2	2	9.5
3	1	3	3	3	8.5
4	2	1	2	3	6.5
5	2	2	3	1	8.0
6	2	3	1	2	9.0
7	3	1	3	2	2.5
8	3	2	1	3	5.0
9	3	3	2	1	4.5
Ave.1	8.17	7.33	7.00	6.83	
Ave.2	7.83	7.50	6.67	6.82	
Ave.3	4.00	5.17	6.33	6.33	
Range	4.17	2.33	0.63	0.50	

Supplementary table S5. The variance analysis

Source of variation	Sum of squares	Degree of freedom	Mean square	F-value	*p*-value	Significance
A（Xanthan gum, mg）	32.1667	2	16.0833	64.3333	0.0153	Remarkable
B（Sodium citrate）	10.1667	2	5.0833	20.3333	0.0469	Remarkable
C（APG）	0.6667	2	0.3333	1.3333	0.4286	
Blank column *	0.5	2	0.25			
Error	0.5	2	0.25			
Sum	43.5					
